# Supplementary material for: A novel system to culture human intestinal organoids under physiological oxygen content to study microbial-host interaction
Source: PLoS One. 2024 Jul 25;19(7):e0300666. doi: 10.1371/journal.pone.0300666 (PMC11271918; doi:10.1371/journal.pone.0300666)
Supplement: S1 File — (DOCX) [file pone.0300666.s001.docx]

**Supplementary Methods.** Detailed protocol for Intestinal Organoid Physoxic Coculture (IOPC) system assembly.

**Preparation (Day before)**

- Autoclave the gaskets, lid (if necessary), and test tubes lids (or another holder) per console.

***Carry out all steps below in a sterile tissue culture hood.***

**Setting up the console**

- Place the magnetic stirrer in the base of the console.
- Open the gas permeable tissue culture plate.
  - Take care not to touch or indent the gas permeable membrane on the base of the plate.
- Using a 10ml syringe filled with vacuum grease and with 200ul pipette tip attached to aid accurate placement, apply vacuum grease to the bottom skirting of the gas permeable tissue culture plate to make ensure an air-tight seal and prevent leaks.
- Plate the gas permeable tissue culture plate base onto the central rubber seal (red).
- Remove the lid from the gas permeable tissue culture plate.
- Place the lid of the console over the top of the gas permeable tissue culture plate.
- Tighten the console by hand using the screws.
- Stick the plastic binding surface of the Silicone-Acrylic Differential Tape (SAD tape) on top of the gas permeable tissue culture plate.
  - Cover half the plate then repeat for the other side. Make sure the tape doesn’t overlap otherwise it won’t be possible to remove the plastic to reveal the sticky surface.
- Using a blade trim off the excess SAD tape from each side of the gas permeable tissue culture plate.
- Taking care to not pierce the lower gas permeable layer of the gas permeable tissue culture plate (don’t put blade too far into the well) and use a scalpel carefully carve out the desired wells (every other well) of the gas permeable tissue culture plate.
  - Using the edge of the well to guide the blade in a circle.
  - Use a checked setup (open every other well) to ensure the gaskets fit.
- Pipette 600ul of differentiation media into each open well.
- Return the lid of the gas permeable tissue culture plate.

**Preparation of the gasket/Transwell**

- Remove film from top layer of the SAD tape to expose the rubber binding surface of the adherent tape.
  - Use common electrical tape to wrap around the outside of the plate – this will create a better seal for the lid and prevent the apical media evaporating.
- Set up the test tubes lids (or other suitable mount) in line to act as mounts for applying the gasket to the Transwell.
- Using sterile forceps transfer a gasket to each test tube (lid).
- By hand, remove Transwell from the 24-well plate and slowly push the Transwell into the gasket.
  - Keep 24-well plate for later when doing the TEER measurement.
- By hand, add each gasket/Transwell in turn to the gas permeable tissue culture plate.
  - Press firmly down to ensure strong binding between red adhesive tape and rubber gasket.
- Add custom lid (see separate methods) to the top of the gas permeable tissue culture plate.
  - Standard gas permeable tissue culture plate can be used but need to be stuck down.

***Carry out all steps below in the anaerobic hood.***

- Attach the gas tube to the inlet and outlet ports.
- Place console on magnetic stir plate and turn onto a low/medium spin.
- Open the cylinder to turn on the flow from the blood gas into the console.
- Set the gas regulator to the left to flow rate 15 for 30 seconds to purge the chamber.
- Then turn the knob to the right to set the long-term flow rate to 0.5 for the remainder of the experiment.
  - Allow system to equilibrate for 2 hours before adding bacterial to the apical media.
- Remove apical media.
- Add 200ul 1:1 BRM and differentiation media to the apical side of the monolayer.
