# Supplementary material for: A novel system to culture human intestinal organoids under physiological oxygen content to study microbial-host interaction
Source: PLoS One. 2024 Jul 25;19(7):e0300666. doi: 10.1371/journal.pone.0300666 (PMC11271918; doi:10.1371/journal.pone.0300666)
Supplement: S2 File — S1 Fig. Intestinal Organoid Physoxic Culture (IOPC) experimental design and modeling. S2 Fig. Measurement of transepithelial electrical resistance under physoxia. S3 Fig. Heat map comparing changes in gene expression of Je HIOs due to physoxia. S4 Fig. Schematic representation of the workflow for microbe HIO co-culture under physoxia. S5 Fig. Changes to gene expression in different Je HIO lines in response to B.thetaiotaomicron co-culture. S6 Fig. Effect of co-culturing B.thetaiotaomicron on physoxia upregulated genes. (DOCX) [file pone.0300666.s002.docx]

**Supplementary Figure 1. Intestinal Organoid Physoxic Culture (IOPC) experimental design and modeling.** (a) Schematic representation of the workflow to prepare Je HIO monolayers in transwells for IOPC experiments. (b) Oxygen consumption models show that organoid monolayers scavenge basolateral oxygen (in red) and prevent leaks into the apical compartment (left panel). The right panel visualizes the gradual dissipation of dissolved oxygen in the absence of an organoid monolayer. (c) Given the oxygen consumption capacity of organoids, calculated time to equilibrium (red arrow) is approximately 2 hours.

**
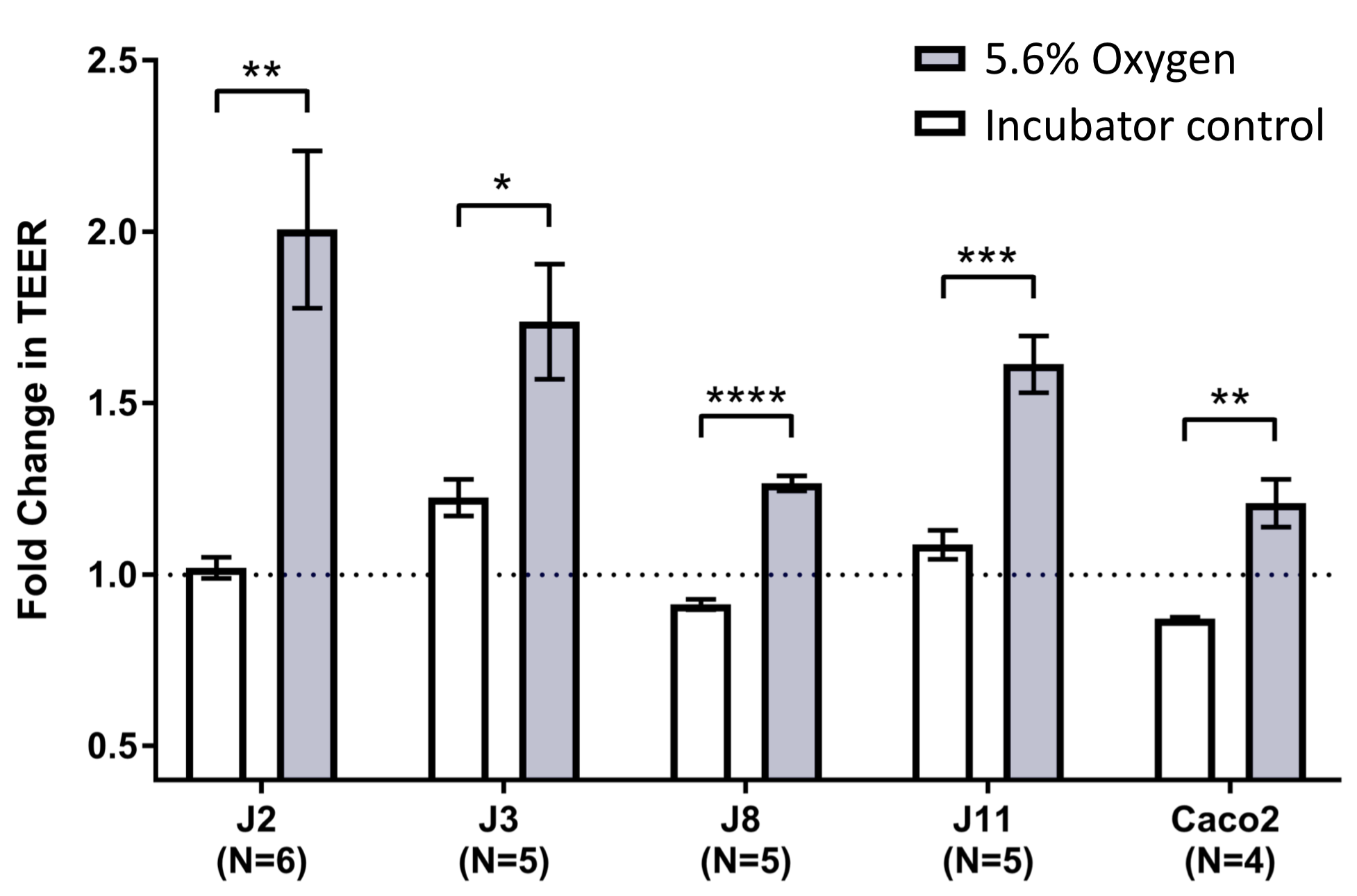
**

**IC**

**IOPC**

**Supplementary Figure 2. Measurement of transepithelial electrical resistance under physoxia.** The phenotype of increased barrier integrity in response to physoxia is seen across all the HIO lines as well as in Caco2 cells. The dotted line indicates the baseline normalized to TEERs from J2 line under standard tissue culture conditions. Error bars reflect standard error; significance determined via unpaired, two-tailed t-test with a Benjamini & Hochberg FDR correction to obtain p-values (* p<0.05, ** p<0.01, ***p<0.001, **** p<0.0001).

­
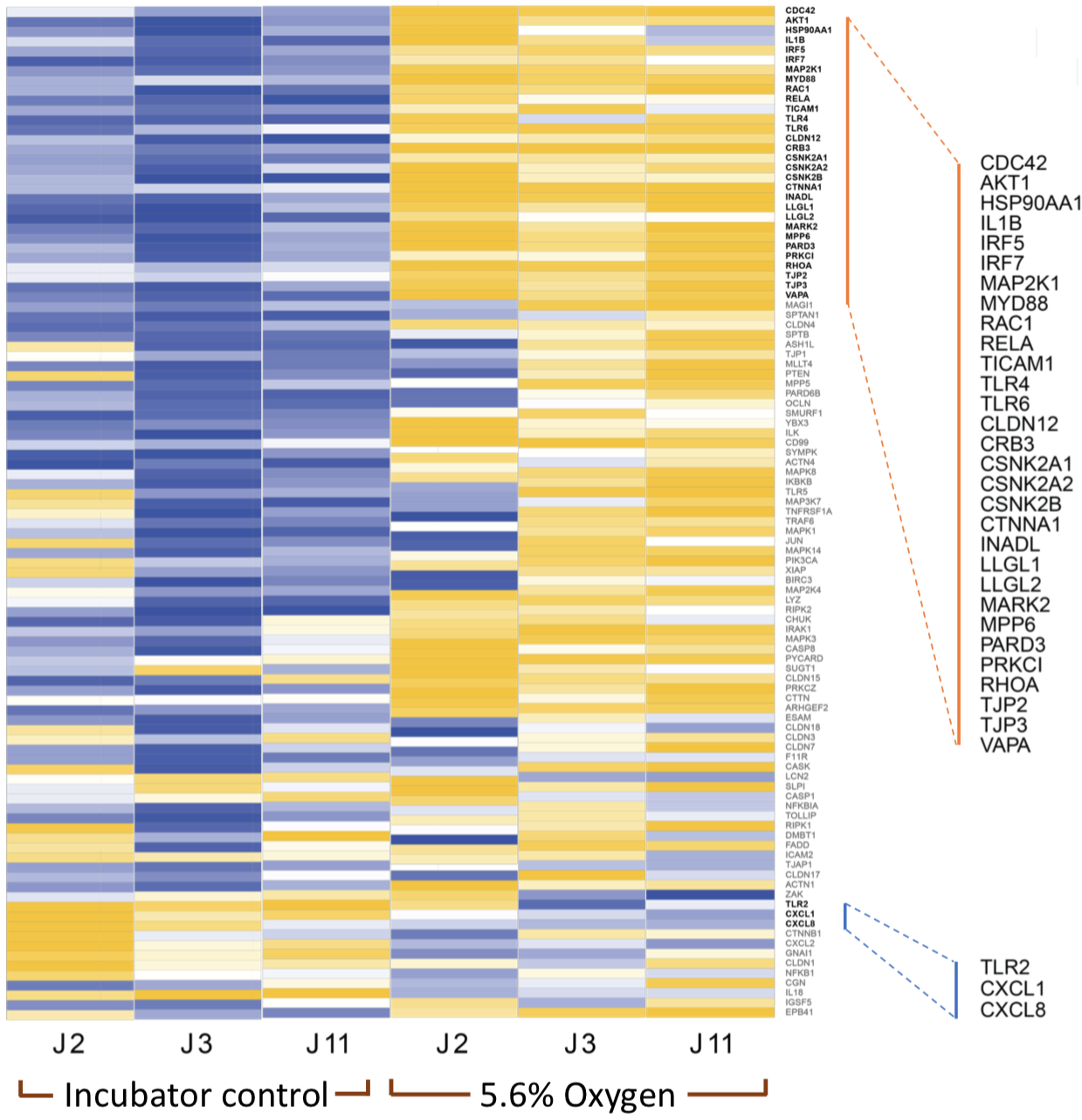


**-1 0 1**


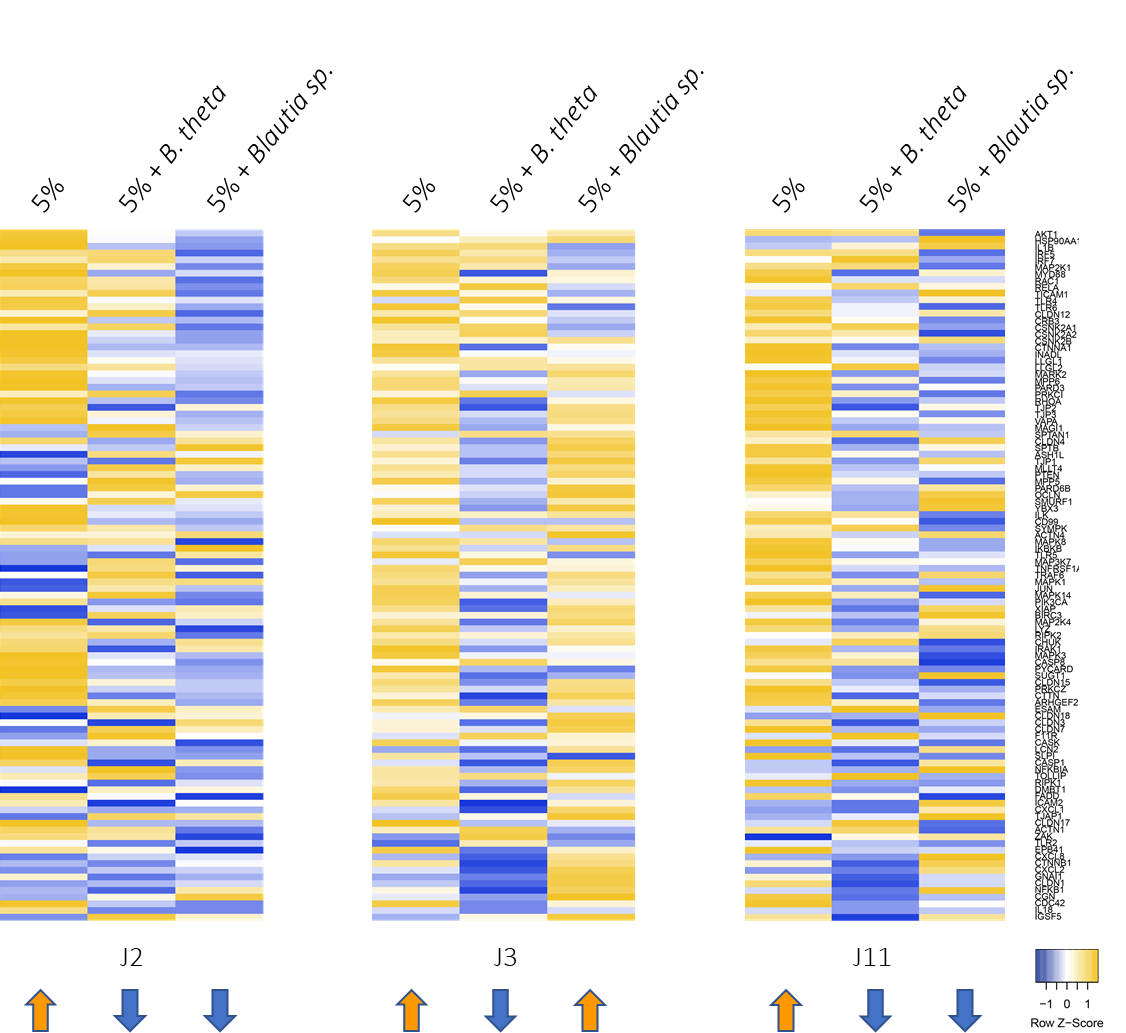


**IOPC**

**IC**

ACTN1

**ACTN1**

**Supplementary Figure 3. Heat map comparing changes in gene expression of Je HIOs due to physoxia.** Gene expression as measured by Qiagen RT2 human tight junctions and human antibacterial panel shows differential regulation of a subset of epithelial integrity and innate immune response genes compared to Incubator control**.** Genes consistently upregulated > 2-fold across all three Je HIO lines in response to physoxia in the orange inset. Genes consistently downregulated >2 fold across all three jejunal lines in the blue inset.

**
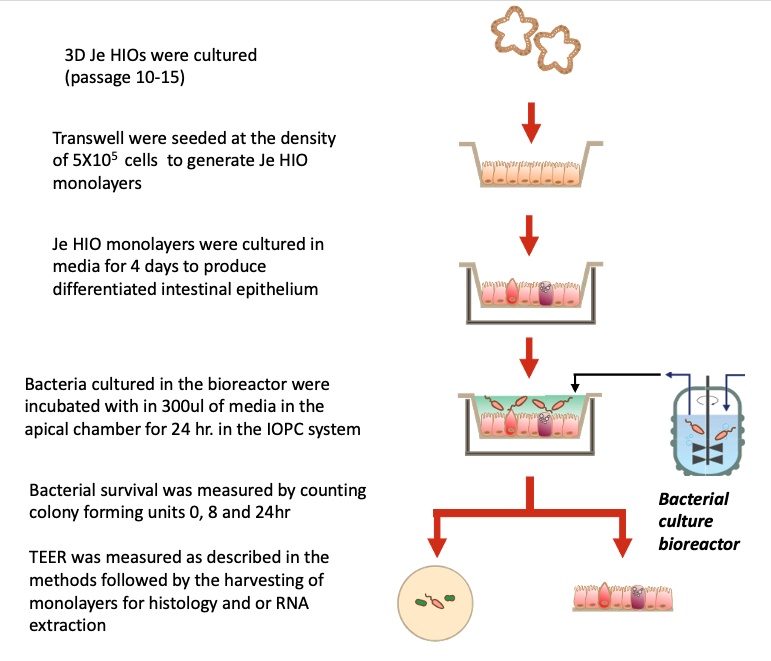
**

**Supplementary Figure 4. Schematic representation of the workflow for microbe HIO co-culture under physoxia.**


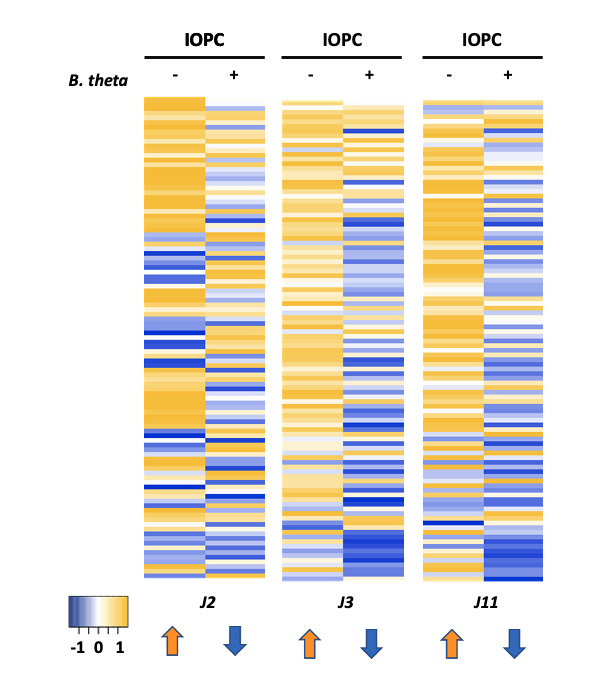


**Supplementary Figure 5. Changes to gene expression in different Je HIO lines in response to B.Theta co-culture.** All three Je HIOs have reduced expression of anti-microbial response and barrier integrity genes in response to *B. thetaiotaomicron*. Gene expression was analyzed by the Qiagen RT2 panel (see legend for Supplementary figure 3 for details)

***B.Theta***

**IOPC**


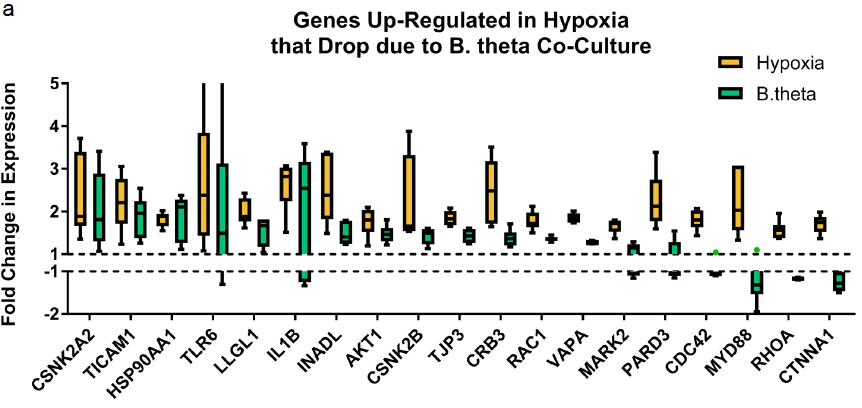


**Supplementary Figure 6. Effect of co-culturing *B. thetaiotaomicron* on physoxia upregulated genes.** Many of the genes that were upregulated in IOPC relative to Incubator control (red dotted line) showed downregulation following coculture to *B. thetaiotaomicron (B. theta*) co-culture.
