# Supplementary material for: A novel system to culture human intestinal organoids under physiological oxygen content to study microbial-host interaction
Source: PLoS One. 2024 Jul 25;19(7):e0300666. doi: 10.1371/journal.pone.0300666 (PMC11271918; doi:10.1371/journal.pone.0300666)
Supplement: Attachment — Submitted filename: Response to reviewer_Fofanova2024.docx [file pone.0300666.s005.docx]

We thank the reviewers for their positive feedback and thoughtful suggestions. We have revised the manuscript to address the concerns raised by the reviewers. Please see our detailed responses below.

1) As suggested by Reviewer 1, we have added the reference ‘Bacterial colonization stimulates a complex physiological response in the immature human intestinal epithelium. Elife. 2017 Nov 7;6:e29132. doi: 10.7554/eLife.29132’ (line 304).

2) Reviewer 1 suggested, “analysis of transcriptional changes in terms of cell subpopulations induced after the contact with bacteria is missing.” We believe such an analysis will need significant optimization and goes beyond the scope of this ‘proof of principle’ manuscript describing a straightforward bacterial-organoid coculture method using off-the-shelf parts.

3) We have modified the legend to IF images in Figure 3c, as suggested by Reviewer 2.

4) Reviewer 2 suggested, “… add some WB to confirm the gene expression in Figure 4”.

Figure 4 shows bacteria-induced differential gene expression in the organoids. Per this suggestion, we conducted western blot analysis for Casp1 and F11R. A widely used Anti-Casp1 showed weak bands at the expected molecular weight for Casp1 and additional bands that haven’t been documented before. On the other hand, a widely used anti-F11R Ab detected multiple bands that have not been reported in previous studies. We believe the discrepancy between our results and previously published studies may reflect differences in the glycosylation pattern between cell lines and intestinal organoids. Based on these results, we conclude that the investigation of gene expression based on western blot analysis needs more significant optimization, including screening of antibodies. Such an analysis is clearly outside of the scope of this manuscript. We strongly believe that the absence of this data does not alter or diminish the coculture method described in this manuscript.

5) Reviewer 2 listed references to be added while discussing the transcriptional changes in organoids. However, we found a reference better suited to discussing transcriptional changes in organoids during coculture with bacteria, so we added that reference (lines 304, 307). The references listed by Reviewer 2 were more appropriate to support the rationale of organoids as a preclinical model (line 350). We appreciate reviewer 2 for pointing out the need to cite relevant references while discussing transcriptional changes during bacterial coculture.

In summary, we firmly believe that the manuscript adequately describes a cost-effective and robust method to coculture intestinal organoids with anaerobic bacteria using ‘off-the-shelf parts’ that will be of immense value to the study of many researchers investigating intestinal microbes.
